# Development and validation of a simulation training platform for the ligation of deep dorsal vein complex in radical prostatectomy

**DOI:** 10.3389/fonc.2024.1407393

**Published:** 2024-10-04

**Authors:** Yu Chen, Qi Tan, Jingzhen Zhu, Luqiang Zhou, Siyue Li, Ji Zheng

**Affiliations:** Army Medical University, Chongqing, China

**Keywords:** laparoscopic simulator, deep dorsal vein complex, radical prostatectomy, low-cost, validation

## Abstract

**Objective:**

This study aimed to design a low-cost, simulation training platform for the ligation of deep dorsal vein (DVC) complex in radical prostatectomy and validate its training effectiveness.

**Methods:**

A simplified prostate urethra model was produced by 0-degree silica gel and pulse pressure banding. This model was placed on a slope of about 30 degrees using cardboard to thus creating a narrow environment of the pelvis. The DVC ligation was performed by a 2D laparoscopy simulator. A total of 27 participants completed the study include 13 novices, 10 surgical residents and 4 urology experts. The novices were trained five trails with 24 hours interval, the residents and experts completed the DVC ligation once. The construct validity of this simulation training platform was performed by completing time, the GOALS (Global Operative Assessment of Laparoscopic Skills) and TSA (i.e. Task Specific Assessments) score. The face validity and content validity were performed by a specific closed-ended questionnaire.

**Results:**

There was no significant difference among three groups in demographic or psychometric variables (*p* > 0.05). Compared to the novices, the residents spend a shorter time to complete the DVC ligation (*p* < 0.05) and had higher GOALS scores (*p* < 0.05), but had no significant difference in TSA scores (*p* > 0.05). Additionally, the experts groups had a better performance compared to residents group in the completing time (*p* < 0.05), GOALS score (*p* < 0.05) and TSA score (*p* < 0.05). The learning curve of novices significantly promoted along with the increased times of training. Almost 90 percent of subjects considered that this simulator had a good performance in the realism and practicability.

**Conclusion:**

We developed a novel low-cost a simulation training platform for the ligation of deep dorsal vein complex in radical prostatectomy, and this simulator had a good performance in the construct validity, face validity and content validity.

## 1 Introduction

Prostate cancer is an epithelial malignant tumor that occurs in the prostate, it seriously endangers people’s health, and its incidence ranks second in male malignant tumors in Europe and the united states, and it also shows a trend of increasing year by year in our country (1). Laparoscopic radical prostatectomy has become one of the best choices for the treatment of prostate cancer, and has shown significant advantages in clinical applications (2). However, the operation is difficult for junior doctors to learn, and the learning curve is long (3), especially for some key surgical steps, the quality of which will directly affect the prognosis of patients (4).

With the development of teaching mode, simulation training has long been proven to be safe and effective in improving the surgical skills of junior doctors and avoid the risks brought by junior doctors to patients in traditional apprenticeship teaching (5, 6). Many simulation training platforms developed by researchers involve various clinical departments, which are convenient, efficient and safe to improve the skills of junior doctors (7, 8). Therefore, the construction of a simulation training platform for key steps of radical prostatectomy is of great significance for junior urological doctors (9). However, the development of simulation training platforms related to laparoscopic radical prostatectomy is very limited at present, and it is mainly aimed at the steps of bladder neck separation and urethra-vesical anastomosis (10). There is almost no development of simulation training platforms for other steps such as ligation of deep dorsal vein complex (DVC) (11). Ligation of the deep dorsal vein complex is one of the key steps in radical prostatectomy, and the quality of its completion will directly affect the patient’s intraoperative blood loss (12). Skilled completion of this step can significantly improve the quality of the operation and speed up the patient’s postoperative recovery (13). Therefore, we developed a simple prostate model and combined it with our previously developed simple simulator to build a simulation training platform for junior urological doctors to practice ligation of deep dorsal vein complex during laparoscopic radical prostatectomy steps and validated the effectiveness of this simulation training platform.

## 2 Materials and methods

### 2.1 The design and production of simulation model

**Figure 1:**
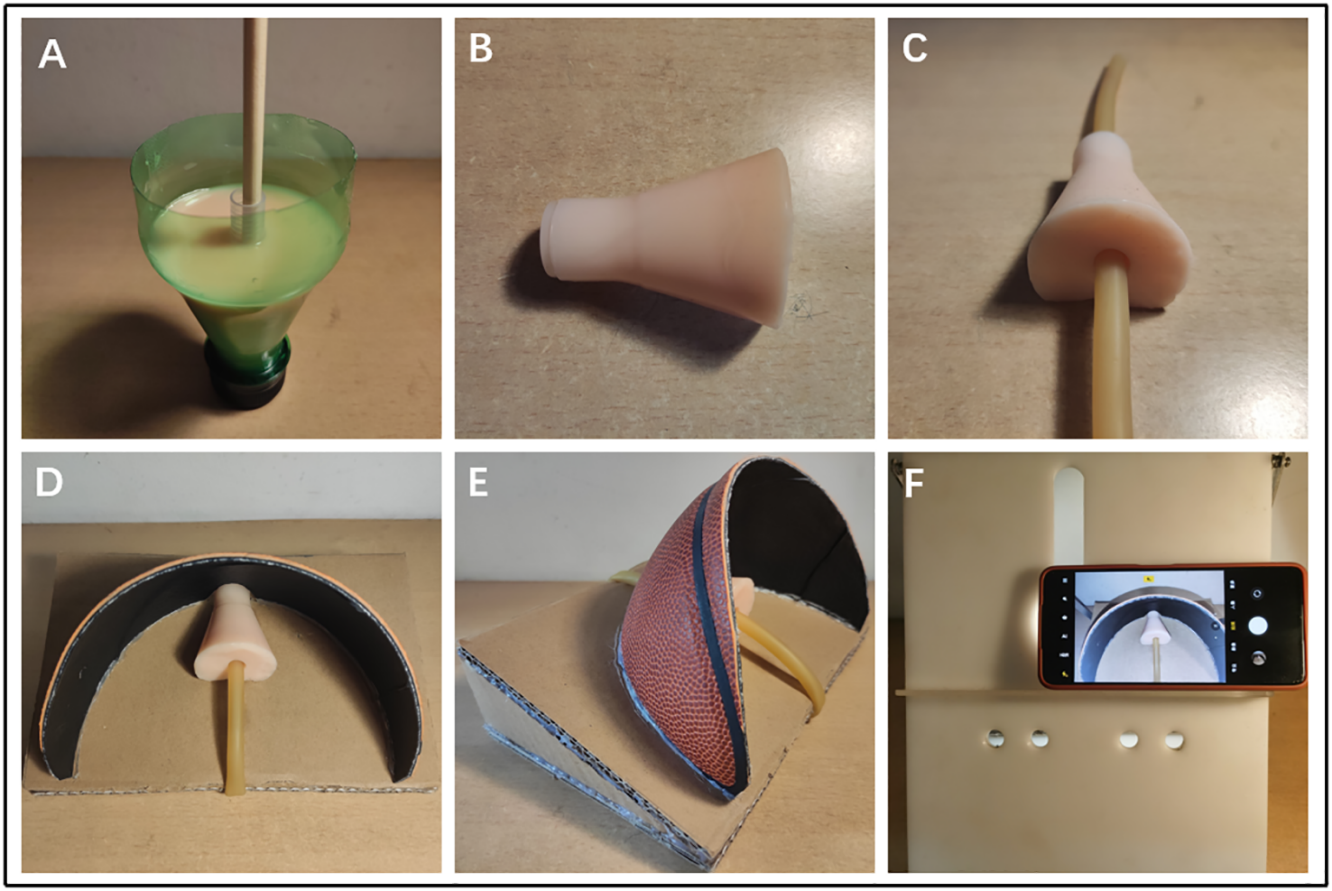
**(A–C)** The production process of prostate and urethra model. **(D–F)** The production process of simulation training platform for DVC ligation.

The design of our prostate model is inspired by the neck of a beverage bottle. A suitable beverage bottle has a neck portion that is similar in shape and size to the prostate. The portion near the mouth of the bottle can also simulate the periurethral tissue at the front of the prostate. Therefore, we use 0-degree silica gel as the material. After solidification, 0-degree silica gel has similar physical properties to prostate tissue. Then, using a suitable beverage bottle as a mold, 0-degree silicone is poured into the beverage bottle, and a plastic pen holder which is used as the urethral channel is fixed in the middle of the model, and its diameter is of about 0.7cm (similar to the urethra). After 48 hours until the silicone gel solidified, the prostate silicone model was carefully removed, and the plastic pen holder in the middle was pulled out to obtain a prostate model with a urethral channel, then we pass the pulse pressure banding through the prostate model to obtain the prostate urethra model, as shown in **Figures 1A–C**.

### 2.2 Construction of simulation training platform for DVC ligation

A simulation training platform was constructed using this simplified prostate urethra model for DVC ligation in laparoscopic radical prostatectomy. We designed a ramp frame with a slope of about 30 degrees using cardboard. Placing the simple prostate model on the ramp frame can imitate the patient’s head-down position, so that the simulated training operation field will be the same as the real operation field of operation. In addition, we cut about 1/4 of the discarded basketball and fixed it on the ramp to simulate the pelvis, thus creating a narrow environment of the pelvis. Finally, we fixed the pulse pressure banding that passed through the prostate model to the ramp frame. We combined the overall model with our previously developed simple laparoscopy simulator (14) to build a complete simulation training platform, as shown in **Figures 1D–F**.

### 2.3 Participants and design

A total of 27 participants completed the study include 13 novices, 10 surgical residents and 4 urology experts, and they are all from the Army Medical University and its affiliated hospitals. The inclusion criteria were as follows: (1) The novices have never console laparoscopic surgery; (2) Surgical residents receiving surgical training have done more than 20 laparoscopic surgical procedures; (3)Urology experts have done more than 50 laparoscopic radical prostatectomy; (4)normal or corrected-to-normal vision, normal stereoacuity. This study was approved by the Southwest Hospital Ethics Committee and the informed consent was signed by all participants.

**Figure 2:**
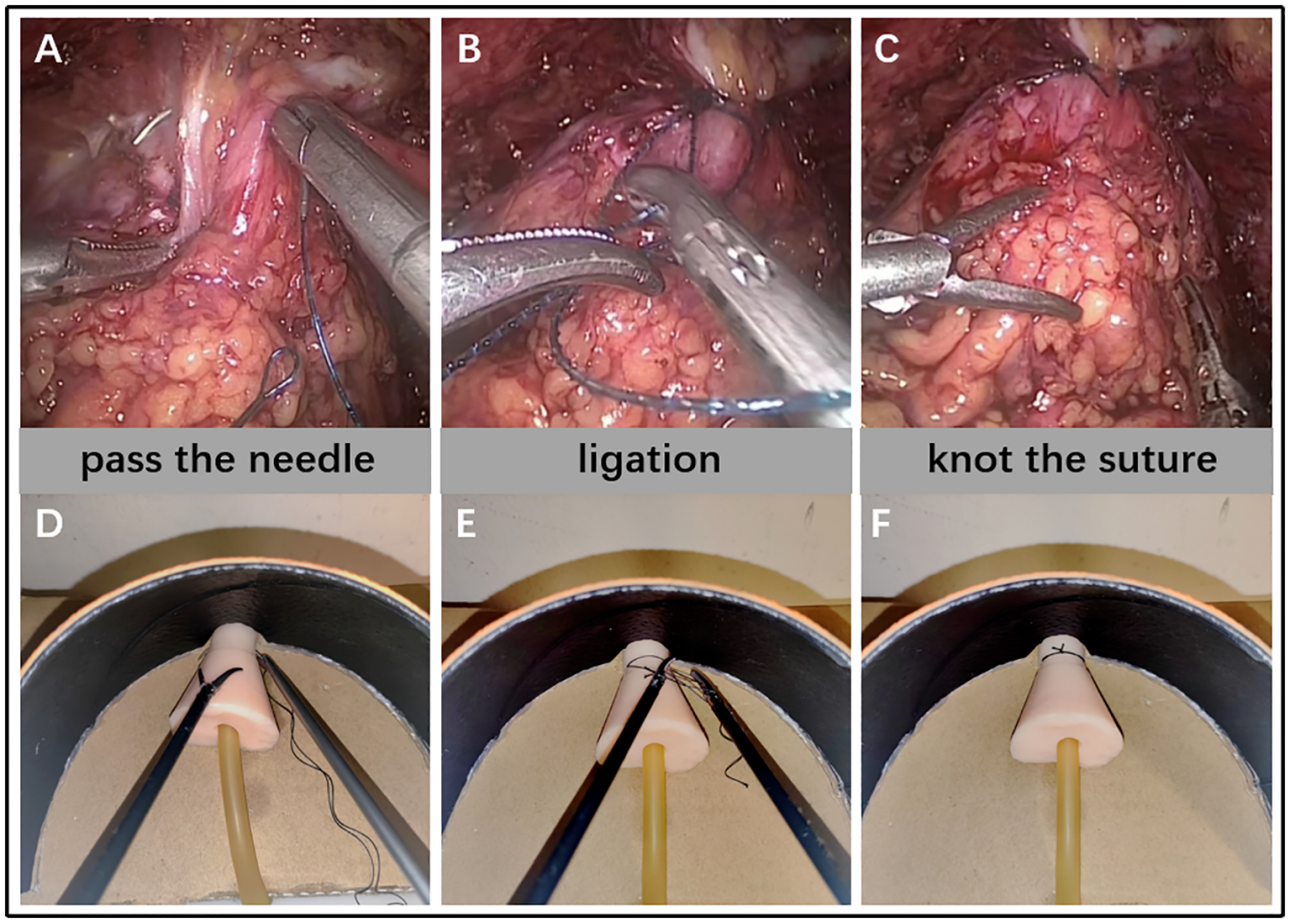
The procedure that surgeon ligated DVC under laparoscopy **(A–C)** VS participants ligated DVC on a 2D simulation training platform **(D–F)**.

Before the start of the test, all participants watched the instructional video of the steps of ligating the DVC, and an urology expert (completed laparoscopic radical prostatectomy>50) explained it, then conducted a teaching demonstration on the simulation training platform developed by us. After that, all participants will perform familiarization exercises on the platform for about half an hour. After the familiarization is completed, each participant starts to operate the professional laparoscopic instruments (including clamps, scissors, needle holders, 2-0 medical suture needles and sutures) on the platform for operational testing, as shown in **Figures 2A–F**. Residents and experts completed only one test, while novices completed five times with more than 24 hours interval between two times.

### 2.4 Validation of the simulation training platform

The construct validity of this simulation training platform was performed by completing time, the GOALS (Global Operative Assessment of Laparoscopic Skills) and TSA (i.e. Task Specific Assessments). According to previous studies (15, 16), the GOALS and TSA were validated for the scale for assessing the operation technique of the DVC ligation, which were assessed by 2 experts and 2 assistants without knowing the identity of each participant. The face validity and content validity were performed by a specific closed-ended questionnaire (5-point Likert scale from 1 = strongly disagree to 5 = strongly agree).

### 2.5 Statistical analyses

The data conformed to normal distribution are expressed as mean ± standard deviation (SD), otherwise expressed as median (IQR). All statistical analyses were calculated using SPSS 25 (2017; IBM Corp, Armonk, NY, USA). Student’s t-test or the Mann-Whitney U test was performed to assess difference between the two groups. A P value of <0.05 was considered statistically significant.

## 3 Result

A total of 27 participants completed the study include 13 novices, 10 surgical residents and 4 urology experts. Demographic experiences are presented in **Table 1**. The expert group had a mean of 5.13 ± 0.85 years’ experience for the radical prostatectomy surgical experience. The Resident group averaged 0.85 ± 0.41 years for the radical prostatectomy surgical experience. Novices had no experience for any surgery.

**Table 1:** Demographical Information.

	Novices	Residents	Experts	*P*-Value
N	13	10	4	
Gender	8M,5G	10M	4M	
Mean Age	20.92 ± 0.76	28.2 ± 1.81	36.5 ± 1.91	**0.000**
Mean Laparoscopic Surgery Experience(years ± SD)	0 ± 0	2.85 ± 1.08	9 ± 1.78	**0.000**
Mean Radical Prostatectomy Surgical Experience(years ± SD)	0 ± 0	0.85 ± 0.41	5.13 ± 0.85	**0.000**

Bold values indicate significant differences in the comparison.

**Figure 3:**
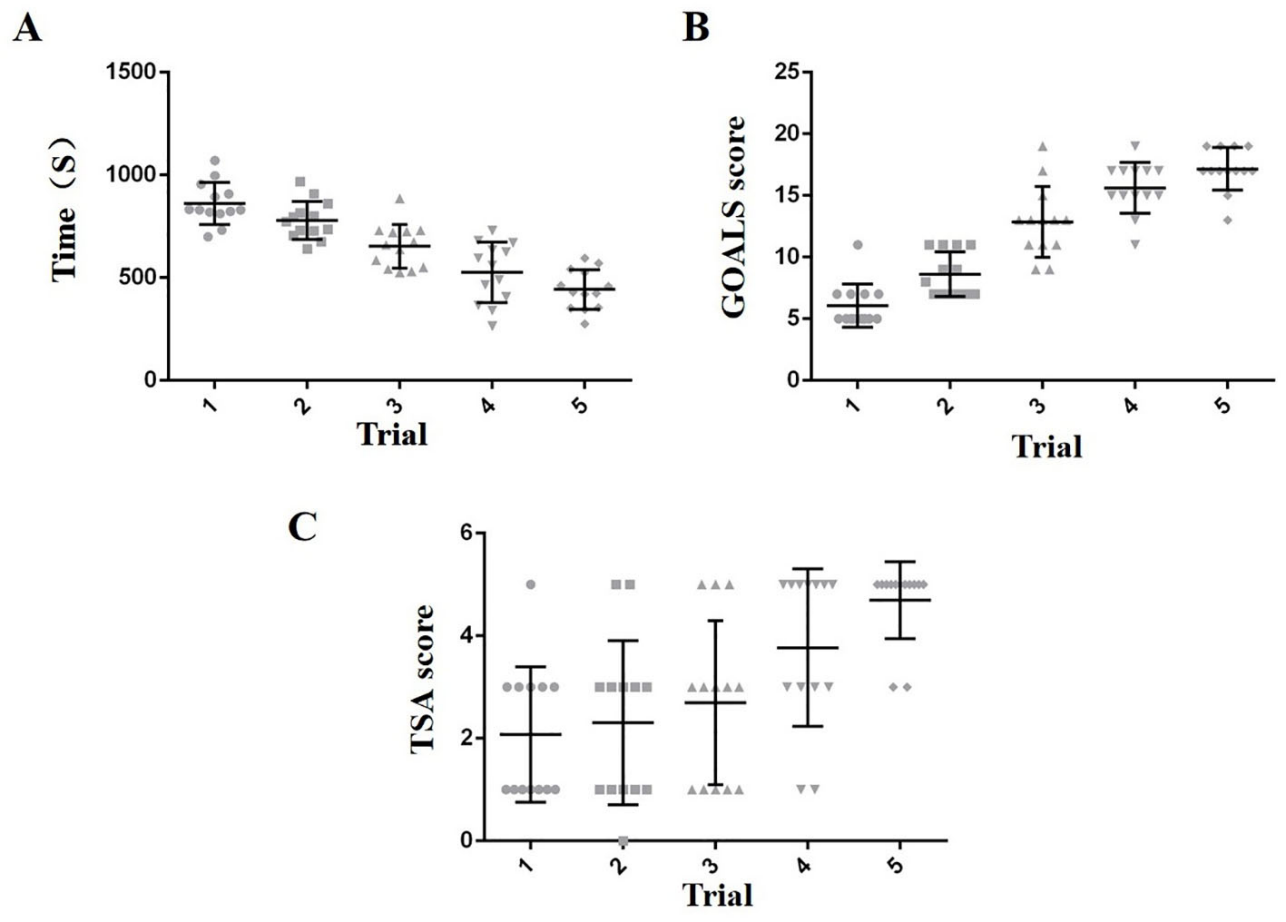
Trend chart of five training results for novices **(A–C)**.

As shown in the **Table 2**, The mean completing times of the first trail of novices was 861.85 ± 102.41s, the median (IQR) of GOALS and TSA score were 5(5, 7) and 1(1, 3). Compared to the novices, the residents spend a shorter time to complete the DVC ligation (*p* < 0.05) and had higher GOALS score (*p* < 0.05), but had no significant difference in TSA score (*p* > 0.05). Additionally, the experts groups had a better performance compared to residents group in the completing time (*p* < 0.05), GOALS score (*p* < 0.05) and TSA score (p<0.05). In order to further assess the training effect of the simulator for novices, the learning curve of novices was performed by 5 trails with 24h interval. As shown in **Figures 3A–C**, the mean completing time was significantly reduced along with the increased times of training, the GOALS score and TSA score were significantly increased along with the increased times of training.

**Table 2:** Construct Validity Results.

	GOALS	TSA	Time (S)
Novices	5(5, 7)	1(1, 3)	861.85 ± 102.41
Residents	21(18.5, 23)	3(1, 3.5)	525.40 ± 71.07
Experts	24(23, 25)	5(5, 5)	398.75 ± 26.07
Novices VS Residents	0.000*	0.284	0.000*
Residents VS Experts	**0.024^#^**	**0.024^#^**	**0.005^#^**

*p < 0.05: novices VS Residents; #*p* < 0.05: Residents VS Experts.

Bold values indicate significant differences in the comparison.

The face validity of simulator was represented by the realism of simulator from user’s judgment and content validity was represented by the practicability of simulator from expert’s judgment. As shown in **Table 3**, almost 90 percent of subjects considered that this simulator had a good performance in the realism and practicability. Majority of participants believe that our simulation training platform can improve the operation level.

**Table 3:** Questionnaire results for the face validity and content validity.

Questionnaire	Strongly Disagree(1)	Disagree(2)	Agree(3)	Strongly
Face validity				
1. Accurately simulates the narrow environment of the pelvis	0 (0%)	2 (7.41%)	11 (40.74%)	14 (51.85%)
2. Accurately simulates the DVC and its surrounding anatomical features	0 (0%)	0 (0%)	17 (62.96%)	10 (37.04%)
3. Accurately simulates the difficulty of ligating DVC	0 (0%)	1 (3.71%)	17 (62.96%)	9 (33.33%)
4. Accurately simulate the operation feel of needle threading and suturing	0 (0%)	5 (18.52%)	15 (55.56%)	7 (25.92%)
Content validity				
5. This is a simple and convenient laparoscopy training platform	0 (0%)	1 (3.71%)	12 (44.44%)	14 (51.85%)
6. It’s a good training tool for ligating DVC for novices	0 (0%)	0 (0%)	10 (37.04%)	17 (62.96%)
7. It’s a good training tool for ligating DVC for experienced	0 (0%)	2 (7.41%)	13 (48.15%)	12 (44.44%)

## 4 Discussion

There is no standardized training system and process in the training and teaching of minimally invasive technology in our country (17). In most teaching hospitals, the teaching method of minimally invasive surgical skills adopts the traditional “apprenticeship” teaching method of teachers and students on the same stage. Due to the lack of pertinence, the learning efficiency in this way is low and the learning cycle is long, and in the early stage of learning, it is easy to lead to increased surgical complications of patients (18). The teaching method of simulation training can make up for the shortcomings of traditional teaching methods (19). Nowadays, various simulation training platforms are flourishing, and cadaver models are usually the “gold standard” for simulation training, which can 100% reproduce real operations, but they are very few in number and complicated in use, making it difficult to be widely used (20, 21). Animal models are also a good choice, which can highly restore the physical properties of tissues and organs, but there are also problems such as cost, complicated use procedures, and difficulty in popularization (22, 23). 3D printing models have become very popular in recent years, it can accurately restore the anatomical characteristics of organs and tissues, however, its high cost and complicated production process discouraged many unpaid medical students (24, 25). Therefore, our research goal is to create a simple simulation training platform with simple manufacturing, low cost and high simulation degree, so that more medical students or doctors can make and use it by themselves, and use this platform to conduct more efficient training for junior doctors, ensuring they safely complete at least one procedure prior to patient surgery, thereby reducing iatrogenic risk to patients.

In the construction of a simple simulation training platform related to prostate cancer, the prostate is one of the important construction organs, and even determines the quality of the overall simulation training platform. Nur Rasyid et al. used ten different proportions of beef and other materials to construct ten tissue models with different physical properties, then they used professional measuring instruments to measure the physical properties of these ten tissue models comparing with real human prostate tissue. Finally they find the tissue model that is closest to the physical properties of the human prostate, and use 3D printing technology combined with the tissue model production method to build a highly realistic prostate model for transurethral prostatectomy training (26). Eunjin Choi et al. also aimed at the training of transurethral prostatectomy training, and constructed a high-fidelity prostate model after mixing different materials. After measuring with professional instruments, it was found that it has similar physical properties to human prostate, and cutting it by electrosurgery has similar performance to cutting the human prostate (27). Although the above-mentioned prostate model has a high degree of simulation, it has disadvantages such as complex construction process, high cost, and poor integration with other organ tissue models, it can only be used for the simulation of specific surgical procedures, such as transurethral prostatectomy training. The silicone prostate model developed by us used the bottle mouth as a mold, most commonly used human silica gel as the perfusion material. Its ingenious design, simple production, low cost, high simulation degree, and it can be better combined with other organs and tissues to build different types of simulation training platforms.so our prostate model construction method is worth popularizing, suitable for medical students and researcher.

We used our prostate model combined with simple models such as pelvis and our original laparoscopic simulator previously developed to build a ligation DVC simulation training platform for the training of DVC ligation steps in radical prostatectomy. DVC is a vascular bundle that exists above the urethra. DVC injury during radical prostatectomy can lead to massive intraoperative bleeding, which can further lead to blurred vision and hinder the progress of surgery. Therefore, efficient intraoperative ligation of DVC is of great significance (28). However, simulation training platforms for DVC ligation steps in radical prostatectomy are rare. Mehrdad Alemozaffar et al. constructed a simulation training platform for key steps of prostatectomy using female porcine genitourinary tract tissue, they used porcine fallopian tube as DVC to simulate ligation of DVC steps, and demonstrated the effectiveness of the platform through novice and expert tests (29). The model uses animal tissue to build a ligation DVC simulation training platform, which is more similar to humans in terms of tissue characteristics, but its female pig reproductive tract is difficult to obtain, the construction process is more cumbersome, the simulation degree of the model shape is low, and there may be ethical issues. The construction materials of the simple ligation DVC platform we built use silica gel, beverage bottles, cardboard, etc., which are easier to obtain, and our production cost will not exceed $10, which is very important for beginners and resident physicians as they do not have a good financial situation. A survey shows that there is very little funding available for training resident physicians in Europe, and I believe this situation may be even more severe in other regions. In addition our model have no ethical issues, and the production process is ingenious and simple. Through measurement, we found that the prostate size, urethral diameter, DVC width and thickness, slope frame angle and other data of our model are very close to the real human situation, so the shape of our model is more simulated and has better face validity. To our knowledge, this is the first simple simulation training platform built for DVC ligation during laparoscopic radical prostatectomy, it will provide new methods for the practice of ligating DVC steps.

We tested our platform to explore its effectiveness by recruiting novices, residents and experts. By comparing novices trial 1,residents and experts, we found that for GOALS scores, novices got a lower score than residents and residents got a lower score than experts. In terms of operating time, novices took more time than residents, and residents took more time than experts, indicating that our platform can differentiate participants in terms of basic laparoscopic skills and complete time. As for TSA scores, novices and residents are not significantly different, and experts performs significantly better than residents, indicating that our platform can differentiate participants in terms of ligating DVC task-specific performance. These can prove that our platform has good construct validity.

Then we conducted five tests on participants in the novices group, their GOALS scores and TSA scores gradually increased, operating time gradually decreased, and through statistical analysis, we found that novices trial 5 had a significant improvement in GOALS scores, TSA scores and operating time than novices trial 1, indicating that our platform can help novices improve basic laparoscopic skills and task completion efficiency.

Finally, all the participants gave a high evaluation to the simulation training platform we developed, proving its face validity and content validity, affirming its role in improving the surgical skills of junior doctors.

Our platform also has some areas for improvement. First of all, although the overall physical characteristics of our prostate model are similar to the real prostate, the details, such as the soft tissue around the urethra, should be softer in the real situation, and the difficulty of puncturing and ligation will be lower. Secondly, the sutures used in real surgery are medical suture needle with thread, and we use medical suture needle that require self-threading for cost and frugal purposes, so the knotting process may be more difficult and time-consuming. Thirdly, our platform mainly trains the step of ligating the deep dorsal vein complex, which is not enough for mastering complex laparoscopic radical prostatectomy, but this basic step can serve as our experience in developing complex steps in the future, we will also continue to explore and research based on the platform’s construction ideas, and build more platforms with other steps, or a multi-step integrated platform. In addition, although robotic prostate cancer surgery has gradually become popular and replaced laparoscopic prostate cancer surgery, our platform seems to have been phased out. However, I believe that laparoscopic technology is the foundation of robotic technology, and practicing laparoscopic operation will also improve robotic operation. Finally, our study suffers from the small sample size, small number of tests, and does not assess whether practice on this model will translate into improved performance in the operating room. Future research is needed to determine whether repeated trainings in our platform lead to better GOALS and TSA scores and reduced patient morbidity.

## 5 Conclusion

Our simulation training platform is a reliable educational tool for junior doctors to learn the steps of ligation of the deep dorsal vein complex in laparoscopic radical prostatectomy. In addition, the model can be combined with GOALS and TSA assessment tools for physicians to objectively assess laparoscopic skill levels before performing procedures on patients.

## Data Availability

The raw data supporting the conclusions of this article will be made available by the authors, without undue reservation.
