# Corrigendum: Development and validation of a simulation training platform for the ligation of deep dorsal vein complex in radical prostatectomy

**DOI:** 10.3389/fonc.2025.1622424

**Published:** 2025-05-21

**Authors:** Yu Chen, Qi Tan, Jingzhen Zhu, Luqiang Zhou, Siyue Li, Ji Zheng

**Affiliations:** Army Medical University, Chongqing, China

**Keywords:** laparoscopic simulator, deep dorsal vein complex, radical prostatectomy, low-cost, validation

In the published article, there was an error in the author list, and author “Yu Chen and Qi Tan” did not mark the co-first author identification. The corrected author list appears below.

“Yu Chen^†^, Qi Tan^†^, Jingzhen Zhu, Luqiang Zhou, Siyue Li and Ji Zheng*

^†^These authors have contributed equally to this work”

The authors apologize for this error and state that this does not change the scientific conclusions of the article in any way. The original article has been updated.

